# The voice of Twitter: observable subjective well-being inferred from tweets in Russian

**DOI:** 10.7717/peerj-cs.1181

**Published:** 2022-12-20

**Authors:** Sergey Smetanin, Mikhail Komarov

**Affiliations:** HSE University, Moscow, Russia

**Keywords:** Subjective well-being, Observable subjective well-being, Happiness index, social networks, User-generated content, Sentiment analysis, Computational social science, Machine learning, Language models

## Abstract

As one of the major platforms of communication, social networks have become a valuable source of opinions and emotions. Considering that sharing of emotions offline and online is quite similar, historical posts from social networks seem to be a valuable source of data for measuring observable subjective well-being (OSWB). In this study, we calculated OSWB indices for the Russian-speaking segment of Twitter using the Affective Social Data Model for Socio-Technical Interactions. This model utilises demographic information and post-stratification techniques to make the data sample representative, by selected characteristics, of the general population of a country. For sentiment analysis, we fine-tuned RuRoBERTa-Large on RuSentiTweet and achieved new state-of-the-art results of *F*_1_ = 0.7229. Several calculated OSWB indicators demonstrated moderate Spearman’s correlation with the traditional survey-based net affect (*r*_*s*_ = 0.469 and *r*_*s*_ = 0.5332, *p* < 0.05) and positive affect (*r*_*s*_ = 0.5177 and *r*_*s*_ = 0.548, *p* < 0.05) indices in Russia.

## Introduction

Social networks have become major platforms of communication and sharing information and opinions (Jakobi, 2017), providing a real-time and rich source of data, including sentiments. Likewise, timely understanding of the sentiment of the population, also defined as *subjective well-being* (SWB), is a of key goal for intergovernmental organizations and governments (Wang et al., 2021) because it not only allows for increasing the speed of the feedback loop for policymakers (Höchtl, Parycek & Schöllhammer, 2016), but SWB can also be considered as a crucial guideline for the development of the state instead of or alongside currently utilised indicators such as gross domestic product (Almakaeva & Gashenina, 2020). Recently, it was found that sharing of emotions oﬄine and online are quite similar (Derks, Fischer & Bos, 2008; Rimé et al., 2020), and researchers began to measure SWB *via* surveys and sentiment analysis of posts from social networks. SWB measured based on automatic processing of digital traces is called *observable subjective well-being* (OSWB), which explicitly characterises the data source as observed (not self-reported) and makes no assumptions about the evaluative or experienced nature of the data (both can be presented in different proportions) (Smetanin, 2022a).

Whereas studies measuring OSWB have already been conducted based on data from other countries (*e.g*., Dzogang, Lightman & Cristianini (2017), Qi, Fu & Zhu (2015), Iacus et al. (2017) and Wang et al. (2021)), research analysing Russian-language content remains quite limited. Several studies (Panchenko, 2014; Shchekotin et al., 2020; Kalabikhina et al., 2021) attempted to measure OSWB for a particular social network or subgroup of the population but did not consider the entire population of Russia and compared the calculated OSWB indexes with survey-based indexes (although this was not necessarily in their scope). To the best of our knowledge, the only study on Russian-language content that both proposed a method for calculating the OSWB index and also compared it with a survey-based index was that (Smetanin, 2022a) based on Odnoklassniki^1^ 1Odnoklassniki is a social network service used mainly in Russia, Belarus, Kazakhstan, and Uzbekistan (Odnoklassniki, 2021). data. Although within that study a high level of correlation (Spearman’s ${r_s} = 0.825$) between the calculated OSWB index and the traditional survey-based OSWB index was achieved, one of the key pitfalls was in the limited data sample: the study considered only 12 months because of Odnoklassniki data availability. Thus, as one of the main future areas of research, it was recommended to calculate the OSWB index over a longer time interval. Moreover, Twitter has often been used for OSWB research in other countries (*e.g*., Dzogang, Lightman & Cristianini (2017), Prata et al. (2016) and Ridhwan & Hargreaves (2021)), but it has not been previously studied in the Russian context. Therefore, the authors were interested to see whether it could serve as a source of Russians’ OSWB data.

In this work, OSWB indicators for Russian-language segment of Twitter^2^ 2Initially, we wanted to focus on Tweets published from Russia, but we realized that this would not work with the chosen data source, so the focus of the study was shifted to Russian-language tweets. were calculated using the Affective Social Data Model for Socio-Technical Interactions (Smetanin, 2022a), which utilises demographic information and post-stratification techniques to make the data sample representative, by selected characteristics, of the general population of a country. The key motivations of this study are to validate whether the OSWB index constructed over a longer period of time (*i.e*., more than 12 months) is a reliable measure of the population’s SWB in Russia and to identify whether Twitter content can be used for measuring SWB. For sentiment analysis, RuRoBERTa-Large (Sberbank, 2021) was fine-tuned on RuSentiTweet (Rogers et al., 2018), and new state-of-the-art results of macro ${F_1} = 0.7229$ and weighted ${F_1} = 0.7281$ were achieved. We calculated several OSWB indices and found that some of them have moderate Spearman’s correlation with the traditional survey-based VCIOM Happiness ( ${r_s} = 0.469$ and ${r_s} = 0.5332$, $p < 0.05$) and VCIOM Positive Affect ( ${r_s} = 0.5177$ and ${r_s} = 0.548$, $p < 0.05$) indices for Russia reported by the Russia Public Opinion Research Center.

The rest of the article is organized as follows. In “Related Work”, related research about sentiment analysis and measuring OSWB based on social networks content in Russia is reviewed. In “Data”, we describe survey-based SWB data and Twitter data. In “Sentiment Analysis”, we document the training of several ML models for sentiment classification of tweets in Russian. In “Observable Subjective Well-Being in Russian-Speaking Twitter”, we outline the approach for measuring OSWB based on tweets. In “Results and Discussion”, we present the results of OSWB measurements and discuss them. In “Conclusion”, we present conclusions from this study and discuss possible future work.

## Related work

According to a recent survey (Smetanin, 2020), there are five categories of applied sentiment analysis studies on Russian-language content categorised by the utilised data source: user-generated content from social network sites, product and service reviews, news from mass media, books, and mixed data sources. We will further focus only on text from social networks as they are relevant to our study. As the most common data source, user-generated content from social networks was studied in three directions: measuring attitudes about different topics (*e.g*., measuring the level of social tension (Koltsova & Nagornyy, 2019) and attitudes towards migrants and ethnic groups (Borodkina & Sibirev, 2019)), identifying specifics of user interaction with content expressing different sentiment (*e.g*., determining the impact of sentiment on the mechanisms of feedback from the audience (Svetlov & Platonov, 2019)), and constructing sentiment indices (*e.g*., measuring well- or ill-being in Russian regions (Shchekotin et al., 2020) and measuring of demographic temperature in pro-natalist and anti-natalist social network groups (Kalabikhina et al., 2021)). When analysing user-generated content from social network sites, one of the key challenges for researchers is the lack of training datasets for specific social networks (Smetanin, 2020). Although several training datasets are available for sentiment analysis of texts from social networks for the Russian language (Kotelnikov, 2021; Smetanin, 2020), only two consist of common-domain texts, are manually annotated, and report inter-annotator agreement scores. The first, RuSentiment (Rogers et al., 2018), consists of general-domain texts from VKontake, and the second, RuSentiTweet (Smetanin, 2022b), consists of general-domain texts from Twitter.

From the sentiment classification perspective, in the past few years, researchers have shifted their focus from rule-based and classic machine learning approaches to approaches based on pre-trained language models to analyse sentiment in Russian-language texts. Fine-tuning of pre-trained language models such as BERT, ELMo, and USE has proven to be one of the most accurate ways to classify sentiment of Russian-languge texts (Smetanin & Komarov, 2021; Kotelnikova, Paschenko & Razova, 2021; Golubev & Loukachevitch, 2021). At present, pre-trained language models have achieved the highest classification results on most Russian-language sentiment analysis datasets available publicly. For example, fine-tuned RuBERT achieved state-of-the-art results on RuSentiTweet (Smetanin, 2022b), LINIS Crowd (Koltsova, Alexeeva & Kolcov, 2016), RuTweetCorp (Rubtsova, 2013), and RuReviews (Smetanin, 2022b) datasets; fine-tuned RuRoBERTa-Large achieved SOTA results on RuSentiment (Rogers et al., 2018). Thus, we fine-tuned a pre-trained language model for sentiment classification in this study.

Recently, the topic of measuring OSWB based on Russian segment of social networks has been widely discussed (Trotsuk & Grebneva, 2019; Shchekotin et al., 2020; Bogdanov & Smirnov, 2021). Several approaches have been proposed for measuring OSWB based on social media data, but in most cases, the indices constructed were not compared with existing SWB indices derived from surveys. For example, Panchenko (2014) constructed an OSWB index based on the Russian-language segment of Facebook by using a rule-based sentiment classification model; Shchekotin et al. (2020) derived an OSWB index from posts of then 1,350 most popular VKontakte regional and urban communities; and Kalabikhina et al. (2021) explored demographic temperature of 314 pro-natalist groups and eight anti-natalist VKontake communities. However, these studies did not compare the obtained results with existing survey-based SWB indices (although in some cases survey data was not available), so it is challenging to verify the reliability of proposed approaches. Also, several studies were dedicated to the development of systems for OSWB analysis of Russian-language content but did not report any results of their analysis. For example, Posevkin & Bessmertny (2015) described an approach to the emotional tonality assessment of public opinion based on sentiment lexicons; Averchenkov et al. (2015) discussed the general concept of social network monitoring through intelligent analysis of text messages; and Sydorenko et al. (2020) proposed a method for classifying time series of tonal ratings based on user posts from social networks. In these studies, only methodological or implementation components were discussed, but none of the indices were reported. To the best of our knowledge, the only study on Russian-language content that both proposed a method for calculating the OSWB index and also compared it with a survey-based index was our recent study (Smetanin, 2022a) based on Odnoklassniki data. Although within that study we achieved a high level of correlation (Spearman’s ${r_s} = 0.825$) between the calculated OSWB index and the traditional survey-based OSWB index, one of the key pitfalls was in the limited data sample: we were able to analyse only 12 months because of Odnoklassniki data availability. Thus, the analysis of longer time intervals remains the relevant research direction for OSWB studies on Russian-language content.

Several articles (Chizhic, 2016; Smetanin, 2017) have been published that consider Russian-language Twitter as a data source for the OSWB, but they were more concerned with the methodology or system for building the index; they omitted description of the resulting OSWB index itself. In the context of measuring OSWB based on the Russian-language Twitter segment, the absence of a significant number of existing studies is quite expected, as the first dataset of general-domain tweets in Russian with manual annotation (Smetanin, 2022b) appeared only in 2022. Thus, studies have not previously published an OSWB index based on the Russian-speaking segment of Twitter and have not compared it with an SWB index based on surveys.

## Data

### Subjective well-being data

**Figure 1:**
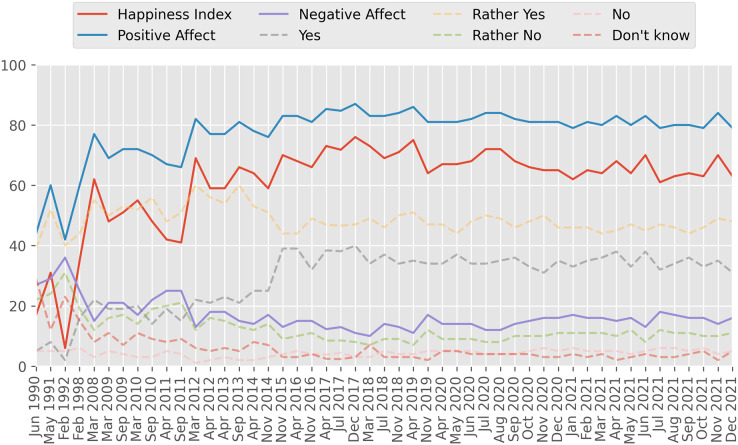
VCIOM Happiness Index. “Life is full of good and bad moments. But in general, are you happy or not?” Close-ended question, 1 answer. Indices are measured in points and can range from −100 to 100. Data source: VCIOM (2022).

SWB in Russia is measured by a series of research organizations (Almakaeva & Gashenina, 2020), such as Russia Public Opinion Research Center (VCIOM), Levada Center, Public Opinion Foundation, the Russia Longitudinal Monitoring Survey of HSE University, the Institute of Psychology of the Russian Academy of Sciences, Ronald F. Inglehart Laboratory for Comparative Social Research of HSE University, and the Center for Sociological Research of Russian Presidential Academy of National Economy and Public Administration. Following our previous study on OSWB in Russia (Smetanin, 2022a), we decided to use the VCIOM Happiness Index because it is available on an almost monthly basis starting from 1990, as presented at Fig. 1. The VCIOM Happiness Index is built on the basis of the following question: “Life is full of good and bad moments. But in general, are you happy or not?” It is calculated as the difference between the sum of positive answers (“Yes”, “Rather Yes”) and negative answers (“No”, “Rather No”). The index is measured in points and can range from −100 (all respondents are unhappy) to 100 (all respondents are happy). The higher the index value, the happier Russians feel. The survey method is a telephone interview of a stratified random sample of 1,600 respondents with landline and mobile numbers. Positive affect and negative affect indices are not explicitly reported by VCIOM, but they can be easily calculated based on their data. Positive affect is calculated as the share of respondents who answered “Yes” or “Rather Yes” on the survey question, and negative affect is calculated as the share of respondents who answered “No” or “Rather No”.

### Twitter data

We selected the Twitter Stream Grab (https://archive.org/details/twitterstream) as a data source of tweets in Russian. Twitter Stream Grab is a publicly available historical collection of JSON content grabbed from the general Twitter “Spritzer” API stream. According to Twitter, this API provides a 1% sample of the complete public tweets and is not tied to specific topics, so we considered it as a good source of general-domain tweets. Additionally, several studies (Wang, Callan & Zheng, 2015; Leetaru, 2019) performed independent validation of the representativeness of this stream. At the time of writing, Twitter Stream Grab included tweets from September 2011 to May 2021 grouped by month; however, some months were missing. VCIOM Happiness was available on a monthly basis from April 1990 to March 2022, but some months were missing too (see Fig. 1). Since our goal was to compare VCIOM Happiness and Twitter-based indices, we selected for further analysis only those months for which both VCIOM Happiness and Twitter data^3^ 3For some months, Tweets were available only for part of the days. We selected only those months for which tweets were available for at least 80% of days. was available. Following this selection strategy, we found 20 months: April 2014, November 2015, April 2016, November 2016, July 2017, March 2018, July 2018, November 2019, April 2020, May 2020, June 2020, July 2020, August 2020, September 2020, October 2020, November 2020, December 2020, March 2021, April 2021, and May 2021.

Considering that the share of geotagged tweets in Russian is extremely low in Twitter Stream Grab (see “Geotagged Tweets in Russia”), we decided to focus on Russian-language tweets in general because they seem to predominantly consist of tweets published from Russia (see “Tweets in Russian and tweets from Russia”). Data were filtered as previously described in Smetanin (2022b). Specifically, since Twitter Stream Grab consists of tweets in different languages, our next step was to remove tweets written in non-Russian languages. Each tweet from this data source already contained information about the language of the text automatically detected^4^ 4Assessing the quality of a Twitter’s language detection algorithm lies outside the scope of this study. Initial research in this direction has already been done in other studies: for example, Pavliy & Lewis (2016) compared the quality of Twitter’s language detection algorithm with Google’s Compact Language Detector on Ukrainian and Russian tweets. The authors found that Twitter’s algorithm correctly detects 92% of texts in Russian and has higher accuracy than Google’s Compact Language Detector. by Twitter, so the language filtering procedure was fairly straightforward. After filtering out all non-Russian tweets and retweets, we obtained 10,869,003 tweets in Russian posted by 1,955,827 unique users (5.55 tweets per user on average) for the selected months.

## Sentiment analysis

### Training data

As the training dataset for the classification model, we chose RuSentiTweet (Smetanin, 2022b), the largest general-domain tweet sentiment analysis dataset in Russian. RuSentiTweet was generated from Twitter Stream Grab—the same data source that we chose to use in our study—so this dataset is ideal for our study. Compared to other available Russian language tweet datasets (Pontiki et al., 2016; Mozetič, Grčar & Smailović, 2016; Lukashevich & Rubtsova, 2016; Loukachevitch et al., 2015; Rubtsova, 2013), RuSentiTweet is the only general-domain dataset of tweets with manual annotation and reported inter-annotator agreement. RuSentiTweet consists of 13,392 tweets annotated into five classes: *Positive*, *Neutral*, *Negative*, *Speech Acts*, and *Skip*. Sentiment classes were previously described in Rogers et al. (2018) and Smetanin (2022b). Specifically, *Positive* and *Negative* tweets represent positive and negative sentiments or attitudes, respectively. *Neutral* tweets are tweets that simply describe a situation in a neutral, factual way and do not contain overt positive or negative sentiment. *Speech Act* tweets perform the functions of various speech acts, such as greeting someone, congratulating someone, or expressing gratitude for something. Although these tweets also express positive sentiment, they are considered a separate sub-category because they can also be made under social pressure or out of a sense of duty (Rogers et al., 2018). *Skip* tweets represent noisy and vague sentiments or attitudes.

### Models

For sentiment classification of Russian texts, approaches based on language models tend to outperform rule-based and basic machine learning–based approaches in terms of classification quality (Smetanin & Komarov, 2021). As reported in the paper presenting RuSentiTweet (Smetanin, 2022b), the SOTA result on RuSentiTweet was achieved by RuBERT (Kuratov & Arkhipov, 2019), a version of multilingual BERT (Devlin et al., 2019) trained on the Russian part of Wikipedia and Russian news. Since RuSentiTweet was published recently (a couple of months ago), so far there are no other works in which classifiers were trained on it—and on which we can rely to select the most effective approach. However, RuSentiTweet was annotated using the same annotation guidelines as RuSentiment (Rogers et al., 2018) (dataset of Russian-language posts from VKontakte), so we can conceivably rely on RuSentiment studies to select potentially efficient models. According to our recent paper (Smetanin, 2022a), RuRoBERTa-Large (Sberbank, 2021) established new SOTA results on RuSentiment and significantly outperformed RuBERT, so we suppose that it may also outperform RuBERT on RuSentiTweet. RuBERT (https://huggingface.co/DeepPavlov/rubert-base-cased) is a version of BERT (Devlin et al., 2019) trained on the Russian part of Wikipedia and Russian news. RuRoBERTa-Large (https://huggingface.co/sberbank-ai/ruRoberta-large) is a version of RoBERTa (Zhuang et al., 2021) model with BERT-Large (Devlin et al., 2019) architecture and the BBPE tokenizer from GPT-2 (Radford et al., 2019) trained on Russian texts. RuRoBERTa-Large is ranked higher that RuBERT in RussianSuperrGLUE (Shavrina et al., 2020) leaderboard (https://russiansuperglue.com/leaderboard/2), so we expected that it will show higher results on RuSentiTweet also.

### Sentiment classification

Fine-tuning of RuRoBERTa-Large was performed using the Transformers library (Wolf et al., 2020) on two Tesla V100 SXM2 32GB GPUs with the following hyperparameters: a number of train epochs from 3 to 5; the number of warm-up steps is 0%, 5%, or 10%; a max sequence length of 128 or 256; a batch size of 18 or 32, a learning rate of 5e−5, an Adam optimiser, and a *softmax* activation function. RuSentiTweet consists of test (20%) and training (80%) subsets, so we used the test subset for computing final classification metrics and the training subset (with additional division to the validation subset) for the model training. We repeated each experiment three times and reported mean values of the measurements.

The best model (four epochs, 10% warm-up steps, 128 max sequence length, 32 batch size) demonstrated macro ${F_1} = 0.7229$ and weighted ${F_1} = 0.7281$, surpassing the existing SOTA results (Smetanin, 2022b) achieved by the RuBERT model (see Table 1). This result was expected because RuRoBERTa-Large is not only higher in the RussianSuperrGLUE (Shavrina et al., 2020) leaderboard (https://russiansuperglue.com/leaderboard/2), but RuRoBERTa-Large also outperformed RuBERT in the sentiment analysis task in our previous study (Smetanin, 2022a). Since RuSentiTweet was released a few months ago, there are currently no other works with which to compare the quality of the classification, but the magnitude of the results generally aligns with the results achieved by other approaches on other five-class sentiment datasets (Smetanin, 2022b).

**Table 1:** Five-class sentiment classification on RuSentiTweet.

Model	Precision	Recall	${\bf F}_{\bf 1}^{{\bf macro}}$	${\bf F}_{\bf 1}^{{\bf weighted}}$
RuRoBERTa-Large	0.7297	0.7248	0.7229	0.7281
RuBERT	0.6793	0.6449	0.6594	0.6675
MNB	0.5867	0.5021	0.5216	0.5189

**Note:**

Results for RuBERT and Multinomial Naive Bayes (MNB) models are from?.

**Figure 2:**
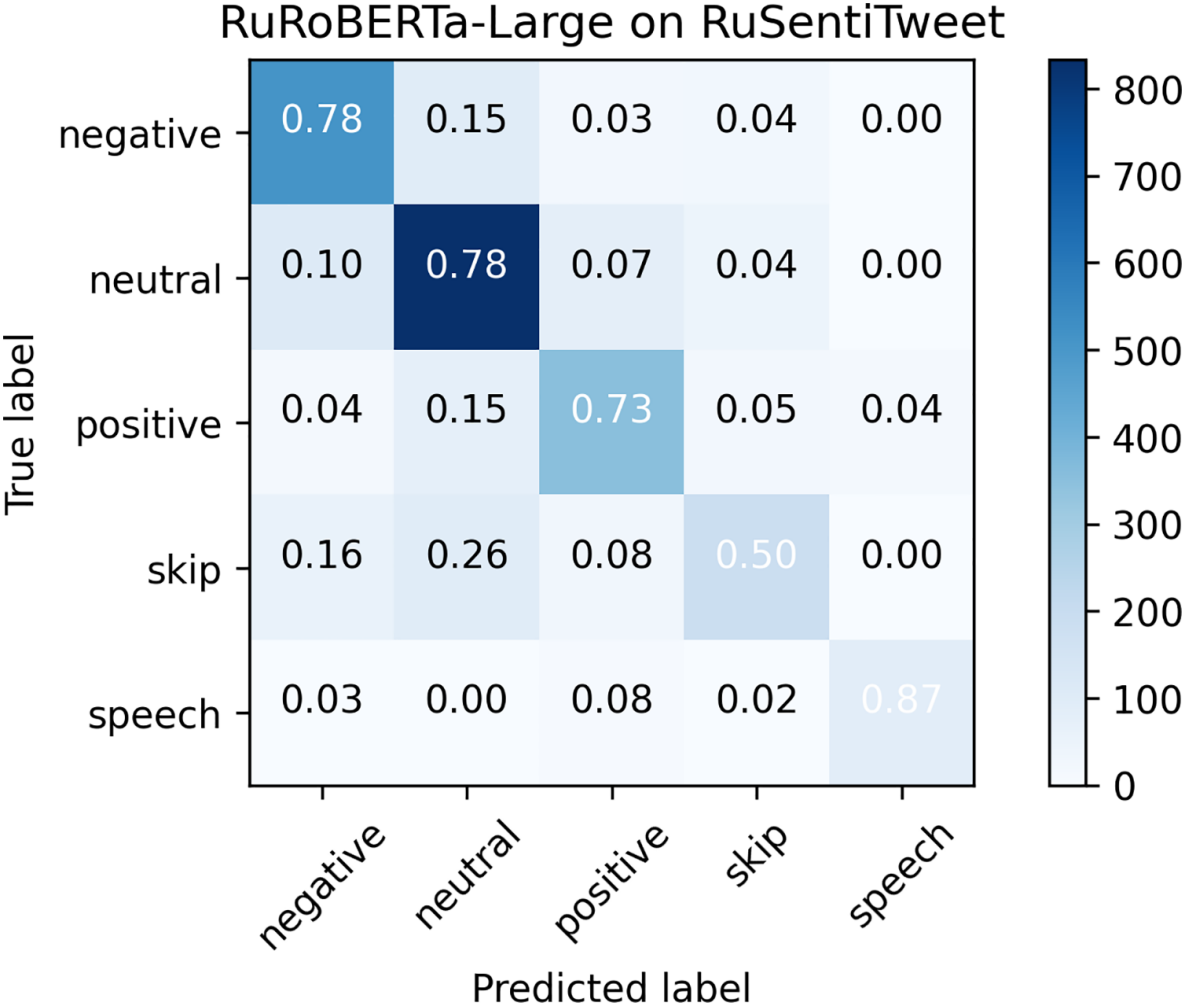
Confusion matrix for RuRoBERTa-Large on RuSentiTweet.

As can be seen from the confusion matrix (see Fig. 2), *Neutral* tweets were commonly misclassified as *Positive* and *Negative*, and vice versa. Also, the *Skip* class was commonly misclassified as *Neutral*. *Speech Act* was clearly separated from other classes, except for *Positive*, because it also represents positive sentiment. In general, the confusion matrix is very similar to that of RuBERT (Smetanin, 2022b). However, the biggest difference is observed in the *Speech Act* class: RuRoBERTa-Large was able to separate it much more efficiently ( ${F_{1,speech}} = 0.8151$) from the rest of the classes than RuBERT ( ${F_{1,speech}} = 0.7444$).

## Observable subjective well-being in russian-speaking twitter

**Figure 3:**
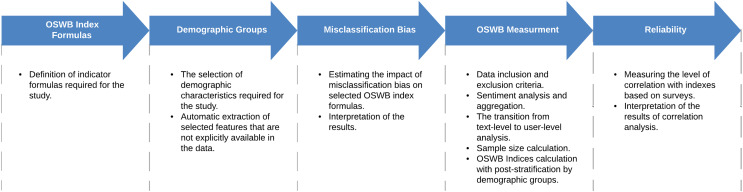
Key steps for measuring OSWB.

To calculate OSWB indicators, we applied the Affective Social Data Model for Socio-Technical Interactions and the approach to calculating OSWB indicators based on texts from social networks proposed in (Smetanin, 2022a). In contrast with Smetanin (2022a), we did not have information about users’ demographic characteristics, so we predicted each user’s gender using a machine learning model. The general pipeline for measuring OSWB based on Twitter data consists of the following steps (see Fig. 3).

1. **OSWB index formulas.** As a first step, it is necessary to define OSWB index formulas of interest. Depending on certain formulas, one of the next steps will evaluate the impact of the misclassification bias on the calculated indices.
2. **Demographic groups.** Next, it is necessary to define demographic characteristics of interest. If some of the defined demographic characteristics are not explicitly presented in the data, then it is also required to automatically extract them. Later identified demographic groups will be used for post-stratification.
3. **Misclassification bias.** Next, it is necessary to estimate the impact of misclassification bias on OSWB indices of interest. Considering that sentiment classification models are predominantly not error-free, they may introduce a bias in the calculated OSWB indices, so it is important to estimate the magnitude of a error that can be introduced by incorrectly classified objects.
4. **OSWB measurement.** This step includes defining data inclusion and exclusion criteria as well as performing sentiment analysis and sentiment aggregation. Then, it is necessary to transition from the text-level to the user-level analysis and check the minimum sample size requirement. Finally, OSWB indices should be calculated with post-stratification by demographic groups.
5. **Reliability.** Once OSWB indices are calculated, it is necessary to measure the reliability by calculating the level of correlation with classical survey-based indices based on surveys.

The detailed description of each step is presented below.

### OSWB index formulas

We selected the following OSWB indicators.

**Definition 1.** $OSW{B_{PA}}$ *is the positive affect indicator (experiencing pleasant emotions and moods) relative to all expressed sentiment and is defined as follows:*

(1) $$OSW{B_{PA}} = \displaystyle{{POS} \over {POS + NEG + NEU + SA + SKIP}}\;OSW{B_{PA}} \in [0;\;1],$$

*where POS is the number of positive posts, NEG is the number of negative posts, NEU is the number of neutral posts, SA is the number of posts with greetings and speech acts, and SKIP is the number of ambiguous posts that cannot be unambiguously assigned to one of the other classes* (Smetanin, 2022a).

**Definition 2.** $OSW{B_{PA,Neu}}$ *is the positive affect indicator (experiencing pleasant emotions and moods) relative to neutral sentiment and is defined as follows:*

(2) $$OSW{B_{PA,Neu}} = \displaystyle{{POS} \over {NEU}},\;OSW{B_{PA,Neu}} \in [0;\; + \infty ]$$

**Definition 3.** $OSW{B_{NA}}$ *is the negative affect indicator (experiencing unpleasant, distressing emotions and moods) relative to all expressed sentiment and is defined as follows:*

(3) $$OSW{B_{NA}} = \displaystyle{{NEG} \over {POS + NEG + NEU + SA + SKIP}},\;OSW{B_{NA}} \in [0;\;1]$$

**Definition 4.** $OSW{B_{NA,Neu}}$ *is the negative affect indicator (experiencing unpleasant, distressing emotions and moods) relative to neutral sentiment and is defined as follows:*

(4) $$OSW{B_{NA,Neu}} = \displaystyle{{NEG} \over {NEU}},\;OSW{B_{NA,Neu}} \in [0;\; + \infty ]$$

**Definition 5.** $OSW{B_{Net}}$ *is the net affect indicator (difference between experiencing pleasant and unpleasant emotions and moods) relative to all expressed sentiment and is defined as follows:*

(5) $$OSW{B_{Net}} = \displaystyle{{POS - NEG} \over {POS + NEG + NEU + SA + SKIP}},\;OSW{B_{NA}} \in [ - 1;\;1]$$

**Definition 6.** $OSW{B_{Net,Neu}}$ *is the net affect indicator (difference between experiencing pleasant and unpleasant emotions and moods) relative to neutral sentiment and is defined as follows:*

(6) $$OSW{B_{Net,Neu}} = \displaystyle{{POS - NEG} \over {NEU}},\;OSW{B_{Net,Neu}} \in [ - \infty ;\; + \infty ]$$

### Demographic groups

Among demographic characteristics, we selected only the gender of the user, whereas in our previous study on Odnoklassniki data both gender and age group were used. Although the author’s age is also an important demographic characteristic that affects SWB in Russia (Rodionova, 2015), we did not extract age from the Twitter data for our study because Twitter is practically unused by the older generations in Russia (Brodovskaya, Dombrovskaya & Sinyakov, 2016). Even if there are some users from the 55+ age group, their share will be extremely small, and as a result, post-stratification by age may negatively affect the index calculation result^5^ 5As was also mentioned in Smetanin (2022a), if some subgroups have either extremely small or extremely large weights, then it can actually make the estimate worse by increasing the model’s variance and sensitivity to outliers (World Food Programme, 2017)..

There was no information about the user’s gender in the Twitter data, so to determine it, we trained a classification model that reached ${F_1} = 0.9835$ in the task of binary gender classification by the user’s full name (see “Gender detection in Russian-speaking Twitter”). We applied this model on Twitter data and found that the distribution of men and women is quite different from the distribution for the Russia’s population. Whereas over the past 10 years Russia’s population was about 46% male and 54% female (Federal State Statistics Service (Russia), 2021), the Twitter users in our data were about 59% male and 41% female.

### Misclassification bias

Given that our sentiment classification model is not error-free, it may introduce a bias in our calculated OSWB indicators. To assess the potential impact of misclassification errors on each of the calculated indicators (see Eqs. (1–7)), we applied the simulation approach for misclassification bias assessment in social indicators research (Smetanin & Komarov, 2022). The core idea of this simulation approach is to simulate the true indicator (*i.e*., real underlying indicator that can be obtained by 100% error-free algorithm); then, on this basis, approximate the results of the classification algorithm using a confusion matrix; and then calculate the quality metrics. We chose Spearman’s correlation coefficients as the main metric and ran 500,000 simulation iterations for each indicator. According to the results of the simulation, the aggregated *p*-values are higher than 0.95, and both coefficients demonstrated almost perfect aggregated correlation scores. According to our results, we did not confirm that our model’s misclassification errors have more than a negligible impact on calculated indicators.

### OSWB measurement

To calculate the indices, we applied the approach described in Smetanin (2022a). The following are summaries of the basic steps for building indices; the full methodology can be found in Smetanin (2022a). When collecting data, we carried out the initial filtering—that is, we left only Russian-language tweets and removed retweets. After filtering out all non-Russian tweets and retweets, we obtained 10,869,003 tweets in Russia posted by 1,955,827 unique users (5.55 tweets per user on average) for the selected months. Next, we transitioned from tweet-level analysis to user-level analysis. To do this, within each analyzed time interval (in our case, within each month), it is necessary to aggregate the sentiment of tweets that each user published. The main motivation for this step is that, first, for post-stratification by demographic characteristics, we need to operate at the level of users, not tweets; second, we need to remove the repeated sharing of emotions associated with the same event, which arises within the phenomenon of social sharing of emotions (Rimé et al., 1991); and thirdly, we need to ensure that tweets published by more active users are not overrepresented in our sample (Németh & Koltai, 2021). We aggregated sentiment on a user level with a majority voting strategy. After that, for each analyzed month, we counted the number of users who published at least one tweet. Although the central idea behind digital data collection for computational social science is collecting as much data as possible (Hox, 2017), it still required verifying that the minimum sample size requirements are fulfilled. The VCIOM Happiness index typically has samples of 1,600 respondents (VCIOM, 2022) for each analysed time period, so we decided to consider this number the minimum sample size for our research. Other research organizations that measure SWB have similar or even smaller sample sizes for each analysed time period and country: for example, FOM The Mood of Others has samples of 1,600 respondents (FOM, 2022), World Values Survey has samples of 1,200 respondents (WWS, 2020), and Standard and Special Eurobarometer surveys typically have samples of 1,000 respondents (GESIS, 2020). We confirmed that for each analysed month, there were more than 1,600 users. Finally, we calculated indices post-stratified by user gender. Information on the gender distribution of the population of Russia was taken from the official statistics of Rosstat.

### Reliability

For reliability measurement, we followed the approach from our previous study on Odnoklassniki data (Smetanin, 2022a) and checked the correlation of OSWB indicators with the VCIOM Happiness Index, VCIOM Positive Affect, and VCIOM Negative Affect. According to previous studies (Stock, Okun & Benito, 1994; Krueger & Schkade, 2008; Levin & Currie, 2014; Kapteyn et al., 2015; Lucas, 2018,), the typical reliability of SWB scales is in the range of 0.50 to 0.84. For single-item SWB measures (like VCIOM measures), the typical reliability is between 0.40 and 0.66 (Krueger & Schkade, 2008). However, considering that our users sample also contains Russian speakers who live outside of Russia (see “Tweets in Russian and Tweets from Russia”) and that age groups were not taken into account during indices calculation^6^ 6In other words, the younger population is over-represented in this sample in comparison with older population., we expected to have a lower correlation than that achieved in our study on Odnoklassniki data (Spearman’s ${r_s} = 0.825$) (Smetanin, 2022a). The results of the reliability measurement are presented in the next section.

## Results and discussion

The correlation^7^ 7Stationarity of time series is violated in accordance with the Augmented Dickey–Fuller Test, so the correlation is calculated based on differentiated time series. between calculated OSWB indices and VCIOM indices can be found in Table 2. Columns without $ASD{M_{STI}}$ represent correlation scores for OSWB indicators calculated without applying $ASD{M_{STI}}$ model (Smetanin, 2022a); that is, they simply count sentiment expressed in the Twitter data. Columns with $ASD{M_{STI}}$ with post-stratification represent correlations scores applying $ASD{M_{STI}}$ model with post-stratification by gender. As can be clearly seen from the results, applying the $ASD{M_{STI}}$ model allows for achieving statistically significant correlation for some indicators. Furthermore, we analyze only the last three columns, in which the model was applied together with post-stratification.

The strongest correlation was found between $OSW{B_{PA,Neu}}$ and VCIOM Positive Affect ( ${r_s} = 0.548$, $p < 0.05$), $OSW{B_{PA,Neu}}$ and VCIOM Net Affect ( ${r_s} = 0.5332$, $p < 0.05$), $OSW{B_{PA}}$ and VCIOM Positive Affect ( ${r_s} = 0.5177$, $p < 0.05$), and between $OSW{B_{PA}}$ and VCIOM Net Affect ( ${r_s} = 0.469$, $p < 0.05$) indices. In general, the values obtained fall within the range of observed reliability of the SWB indicators reported in other studies. However, in the context of estimating the Russian population’s SWB in the manner described in this article, we believe that Russian-language tweets from Twitter Stream Grab can be used as additional information to the traditional survey-based SWB indicator—but not as the main source of information. The main reason, in our opinion, is that based on the available data, we cannot reliably distinguish Tweets published from Russia and also take into account the opinions of all age groups. Moreover, these OSWB indicators seems to be more representative of the younger age groups than they are the main Twitter audience in Russia. However, given that a statistically significant correlation was obtained even on these noisy data, we assume that with access to a larger volume of tweets from Russia, we can obtain an even stronger correlation and potentially prove that Twitter can be used on its own as a reliable source of data on OSWB.

**Table 2:** Spearman’s correlation between OSWB and VCIOM indices. Bold entities indicate statistically significant results.

Index	Without ${\bf ASD}{{\bf M}_{{\bf STI}}}$			With ${\bf ASD}{{\bf M}_{{\bf STI}}}$		
	VCIOM Net	VCIOM PA	VCIOM NA	VCIOM Net	VCIOM PA	VCIOM NA
$OSW{B_{PA}}$	0.0167	−0.0241	−0.1027	0.469**	0.5177**	−0.2602
$OSW{B_{PA,Neu}}$	0.0458	−0.0062	−0.1168	0.5332**	0.548**	−0.3292
$OSW{B_{NA}}$	−0.0211	−0.1497	0.0053	0.3476	0.2602	−0.262
$OSW{B_{NA,Neu}}$	−0.1117	−0.1372	0.092	0.366	0.3012	−0.2496
$OSW{B_{Net}}$	0.0854	0.1809	−0.115	−0.1109	0.0321	0.1841
$OSW{B_{Net,Neu}}$	0.0458	0.1622	−0.0602	−0.0933	0.0624	0.1664

**Note:**

VCIOM Net is VCIOM Happiness Index, VCIOM PA is VCIOM Positive Affect, and VCIOM NA is VCIOM Negative Affect. Symbol ** indicates *p*-value less than 0.05.

Even though we obtained a statistically significant correlation between our OSWB indices and VCIOM indices, it was less significant than in the study based on Odnoklassniki data (Smetanin, 2022a). We assume that this is due to the following factors. First, our dataset of tweets contained some tweets that were not from Russia (see “Tweets in Russian and Tweets from Russia”). A Russian-speaking population not from Russia may have a different level of SWB and, as a result, could influence the resulting OSWB indices of our study in one direction or another. Second, older age groups were not sufficiently represented in our study, whereas in the study based on Odnoklassniki data they were presented and also taken into account at the post-stratification stage. Since the level of SWB in Russia, among other things, depends on age (Rodionova, 2015), this also influenced our OSWB indices. We believe that if more age groups were more evenly distributed in the Twitter data, then the correlation between our OSWB indices and the VCIOM indices would increase. Third, VCIOM respondents are aged 18 and over, whereas according to Twitter policy in Russia, people aged 14 and over can use it, so our data partially covers people aged 14 to 17—who are not included in the VCIOM surveys. Fourthly, we assume that perhaps in Russia Twitter is not as good a source of data for OSWB research as Odnoklassniki. As was highlighted in Smetanin (2022a), even though the emotional communication online and oﬄine is surprisingly similar (Derks, Fischer & Bos, 2008; Rimé et al., 2020), it has been found that social norms, social media platform characteristics, and individual preferences may influence social network choice for sharing a particular type of emotion. In particular, Vermeulen, Vandebosch & Heirman (2018) confirmed that Facebook statuses, Snapchat, and Instagram are mostly used for sharing positive emotions, whereas Twitter and FB Messenger are also used for sharing negative emotions. As a hypothesis, emotions expressed in the Russian-speaking Twitter are perhaps as not as associated with the SWB level as in the case of Odnoklassniki. Interestingly, no statistically significant correlation was found between the expression of negative emotions ( $OSW{B_{NA}}$ and $OSW{B_{NA,Neu}}$ indicators) and the VCIOM indices. This result also coincides with the result of our previous study based on Odnoklassniki data (Smetanin, 2022a), since there was also no statistically significant relationship found there. As a hypothesis, it can be assumed that the manifestation of negative emotions in social networks is not associated with classical positive affect, negative affect, nor net affect measures.

## Conculsions

In this work, we calculated OSWB indices for the Russian-language segment of Twitter and confirmed moderate correlation between two of calculated indices ( $OSW{B_{PA}}$, $OSW{B_{PA,Neu}}$) with the survey-based indices reported by VCIOM. The contribution of this study is fourfold. First, to the best of our knowledge, this is the first study to have reported OSWB indices for the Russian-language segment of Twitter and obtain statistically significant correlation with the survey-based indices. Second, we demonstrated the importance of applying $ASD{M_{STI}}$, as it allows us to achieve the moderate level of correlation with survey-based indices. Third, we achieved new SOTA results of ${F_1} = 0.7229$ on RuSentiTweet and made the model publicly available. Lastly, we presented an approach for gender detection based on Russian names with ${F_1} = 0.9835$ and made the model publicly available.

Future studies on the current topic are therefore recommended. First, it would be interesting to build an OSWB index based on data from the social network VKontakte since it is the most popular in Russia and has the largest audience. Second, it would be interesting to use a more detailed breakdown into specific emotions that appear in texts to build the OSWB index. Third, methods for accounting for contextual messages (such as comments or retweets) should be explored, as they can also contain valuable information for building the OSWB index. Fourth, other digital traces, such as user search queries, can also be considered as a data source for constructing an OSWB index. Finally, the strategies for recommended sample size calculation for social indicators research based on digital traces can be investigated.

## Appendix

### Geotagged tweets in Russia

Initially, we planned to use geotagged tweets to select those that were published in Russia but found two major concerns with this approach. First, we approximately estimated the share of geotagged tweets in Russia based on existing data sources and found that this number is too small for conducting OSWB study. Considering that Twitter generates around 200 billion tweets per year (Gao et al., 2022), only around 373.2 million tweets are generated in Russia (Brand Analytics, 2021), approximately 0.85% of tweets are geotagged (Sloan et al., 2013), and Twitter Stream API provides approximately 1% sample of tweets, we can estimate that the share of geotagged tweets from Russia is approximately 0.0000013, and Twitter Stream API can provide around 220 tweets from Russia per month, which is too small for OSWB study. Second, Sloan & Morgan (2015) found that there are significant demographic variations between those who opt in to geoservices and those who geotag their tweets and suggested that Twitter users who publish geographical information are not representative of the wider Twitter population.

### Tweets in Russian and tweets from Russia

Since in this study we are comparing OSWB indicators with the VCIOM Happiness Index for Russia but the OSWB indicators are based on Russian-language tweets (and not tweets from Russia), the question arises of what proportion of tweets in Russian are actually published from Russia. According to various estimates (Arefyev, 2013; BusinesStat, 2021; Kaganov, 2021), most of the Russian-speaking population lives in Russia, and this proportion is growing over years. Moreover, whereas almost all (95.76%) of Russian-speaking population in Russia use Russian as a language of communication, less than a half (45.54%) of Russian speakers outside Russia do so (BusinesStat, 2021). Based on these data, we approximately estimated the share of Russian speakers who use Russian as the language of communication starting from 2004 (see Table 3). If we assume that if a person uses Russian as a language of communication then the person also communicates in Russian on Twitter, then we can assume that in 2004 68% of Russian-language tweets were published from Russia, and by 2019 this number increased to 73.7%. However, it is worth noting that this estimate is rather superficial and does not take into account other important factors, such as the level of internet penetration and Twitter penetration in each country. Thus, it seems that filtering by language without filtering by location allows selecting almost all users from Russia; however, about a quarter of Russian-speaking users who do not live in the territory of Russia are also presented in the sample. This estimation indicates that we should not expect high correlation between OSWB indicator based on Russian-language tweets and VCIOM indices.

**Table 3:** Geographical distribution of the Russian-speaking population.

Year	Russian Speakers			Russian as the Language of Communication		
	World, M	Russia, M	Russia, %	World, M	Russia, M	Russia, %
2004	278	140	50.36	196.90*	134.06*	68.09*
2010	259.8	137.5	52.93	187.36*	131.67*	70.28*
2015	243.1	137	56.36	179.51*	131.19*	73.08*
2019	256	146.2	57.11	190	140	73.68

**Note:**

The number of Russian speakers is from Arefyev (2013), BusinesStat (2021) and Kaganov (2021). The number of Russian speakers who use Russian as the language of communication for 2019 is from BusinesStat (2021). The * symbol denotes that this value was calculated using an estimation that 95.76% of Russian speakers who live in Russia use Russian as a language of communication and 45.54% of Russian speakers who live outside of Russia use Russian as a language of communication (BusinesStat, 2021).

### Gender detection in Russian-speaking Twitter

Previous studies on gender detection of Russian social media users have shown that depending on the source of data from which gender is determined, the quality of the classification changes significantly. For example, one of the tasks of RusProfiling PAN at FIRE Track competition (Litvinova et al., 2017b) was to to classify the gender of tweets in Russian based on their text. The best system (Markov et al., 2017) reached only 68.25% accuracy in binary classification, and the organizers of the track hypothesised (Litvinova et al., 2017b) that the problem was that tweets are shorter and grammatically poorer in comparison with other sources (*e.g*., Facebook posts or essays). There were other studies on text-based gender detection that reported higher results, such as Sboev et al. (2019), which showed 0.62 accuracy on LiveJournal posts; Litvinova et al. (2017a) achieved accuracy of 0.72 on tweets and 0.71 on Facebook posts; and Bogachev et al. (2020) reported ${F_1}$ of 0.84 on RusPersonality (Litvinova et al., 2016). Besides linguistic characteristics, some studies also considered information from user profiles as well as social graph. For example, Tolmachev (2019) achieved accuracy of 0.715 by utilising social graph information. At the same time, it seems that the task of gender detection for Russian social media users tends to be much easier if there is access to the user’s full name. Panchenko & Teterin (2014) showed on a dataset of 100,000 Russian full names from Facebook that simple and computationally efficient models (*e.g*., n-grams with Logistic Regression) yield excellent results and are able to achieve accuracy up to 96%. The high accuracy of classification based on the full name is quite expected because, first, in the Russian language, first names are usually clearly divided into male and female, and second, the surnames can commonly be attributed to one of the genders based on endings. Thus, considering that our data source contains full names of Twitter users, we decided to use an approach that relies only on the full name for gender detection.

Since the datasets used in existing works for gender detection are not publicly available, we created our collection of full names and genders based on data from Russian social networks. It is not possible (at least now) to automatically create this collection from Twitter because the Twitter API does not provide information about a user’s gender. Thus, we decided to focus on data from VKontakte—the largest Russian online social media and social networking service, which is predominantly used by Russian speakers. In VKontakte, we selected the top-1 group (https://vk.com/public27895931) based on Medialogia rating (Medialogia, 2021) and loaded all the users with specified gender *via* VKontakte API (https://dev.vk.com/method/groups.getMembers). We collected 13,126,794 VKontake profiles, where each profile contains gender and first and last names written in Cyrillic and Latin alphabets. We did not consider users with unknown gender. Among these profiles, we obtained 6,521,854 unique full names in the Cyrillic alphabet and 6,263,813 unique full names in the Latin alphabet. The number of full names in the two alphabets differs due to the fact that different names in the Cyrillic alphabet can have both the same and different transliteration into the Latin alphabet, and even similar names in one alphabet can have different transliteration to another alphabet. Based on the data obtained, we formed the final dataset for training models using the following logic: if the user’s name in Latin and Cyrillic is different, we added both names to the dataset; if they are the same, we added only one name. The final dataset contains 25,101,673 names (46% male and 54% female). Interestingly, the distribution of male and females in our dataset almost perfectly matches the male and female distribution of population in Russia: over the past 10 years, the proportion of males has been around 46% males and 54% females (Federal State Statistics Service (Russia), 2021).

Following the approach by Panchenko & Teterin (2014), we used L2-regularized Logistic Regression with character *n*-grams to classify gender. In order to identify the best hyper-parameters (*e.g*., character n-grams type, n-grams range, usage of IDF, TF-IDF normalisation type), we first ran a grid search with 10-fold cross-validation (80%—training subset, 20%—test subset) on a random sample of 100,000 full names. The model with character n-grams inside word boundaries, n-grams range of (2, 7), usage of IDF, L2 TF-IDF normalisation, and ignoring terms that appear in more than 50% of the documents showed the best ${F_1}$ score of 0.9771, so we used these hyper-parameters to train the final model on the whole dataset. The final model trained in the full dataset demonstrated ${F_1} = 0.9835$ on the test subset (20% of full names). The magnitude of our results is in line with the results achieved by Panchenko & Teterin (2014) (96%) on Russian full names from Facebook, which can be explained by the fact that Russian full names commonly contain grammatical information about the gender. Finally, we applied this model to names from Twitter to classify users’ gender.

### Holidays in Russain-speaking Twitter

Considering that positive emotions expressed in social media have stable fluctuations during different time periods, such as weekly (Dzogang, Lightman & Cristianini, 2018; Smetanin, 2022a) and monthly variations, we decided to focus only on speech acts and greeting as the proxy measure of the popularity of holidays because so far there has been no evidence that they also have strong fluctuations.

**Definition 7.** $OSW{B_{SA}}$ *is the the share of greetings and speech act tweets relative to relative to all expressed sentiment and is defined as follows:*

(7) $$OSW{B_{SA}} = \displaystyle{{SA} \over {POS + NEG + NEU + SA + SKIP}},\;OSW{B_{SA}} \in [0;\;1]$$

Taking into account that there is also some evidence that people of different genders may have different attitudes towards different holidays (VCIOM, 2018), we decided to first calculate the holiday rating for each gender separately, and then build an aggregate rating.

The results of the analysis are presented in Table 4.

**Table 4:** Greetings and speech acts on Russian-speaking Twitter during holidays.

Holiday	Date	Male			Female			Rank, All days	Rank, Holidays
		$OSW{B_{SA}}$	Rank, All days	Rank, Holidays	$OSW{B_{SA}}$	Rank, All days	Rank, Holidays		
New Year	2020-12-3	0.1192	1	1	0.1290	1	1	1	1
Defender of the Fatherland Day	2020-02-23	0.0599	4	3	0.0855	2	2	2-4	2-3
International Women’s Day	2020-03-08	0.0666	3	2	0.0757	3	3	2-4	2-3
New Year	2020-01-01	0.0483	7	4	0.0562	12	6	7-8	4
Victory Day	2020-05-09	0.0425	12	7	0.0577	8	4	9	5
Halloween	2020-10-31	0.0448	11	6	0.0569	11	5	10	6
Knowledge Day	2020-09-01	0.0451	10	5	0.0490	19	7	12	7
Easter	2020-04-19	0.0402	23	8	0.0485	22	8	16	8
Christmas	2020-12-25	0.0391	30	10	0.0464	35	11	19	9
Eid al-Adha	2020-08-02	0.0392	28	9	0.0426	100	14	38	10
Russia Day	2020-06-12	0.0347	115	13	0.0451	50	12	62	11
Eid al-Adha	2020-07-31	0.0335	151	14	0.0473	30	10	72-73	12
Eid al-Adha	2020-08-03	0.0321	192	17	0.0476	26	9	97-99	13
Unity Day	2020-11-04	0.0335	153	15	0.0420	115	15	132	14
International Workers’ Day	2020-05-01	0.0318	199	18	0.0436	72	13	135-136	15
Eid al-Adha	2020-08-01	0.0350	104	12	0.0384	193	17	152-154	16
Cosmonautics Day	2020-04-12	0.0359	79	11	0.0325	287	20	202	17
Eid al-Adha	2020-07-30	0.0305	232	19	0.0407	140	16	205	18
Valentine’s Day	2020-02-14	0.0323	185	16	0.0383	198	18	212	19
Christmas	2020-01-07	0.0273	275	21	0.0365	230	18	257	20
Chinese New Year	2020-01-25	0.0304	237	20	0.0322	289	21	267-268	21
Saint Patrick’s Day	2020-03-17	0.0235	329	22	0.0283	336	22	340-341	22

**Note:**

The list of holidays is based on VCIOM (2018).

- **Official public holidays.**

– The New Year is the most popular holiday on Russian-speaking Twitter: the share of greetings and speech acts on December 31 exceeds the average annual value by more than three times and reaches 12.3% of all tweets for that day. The share of greetings and speech acts decreases on January 1 to 5.3%, but is still quite high and ranks 7th among all days of the year. This observation is fully consistent with the VCIOM (2018) survey, in which the new year was also the most popular holiday (celebrated by 96% of people), and with the study by Volovikova (2018), where it is listed as a favorite holiday for all groups.
– Defender of the Fatherland Day and International Women’s Day share the second and third places in the ranking of the most popular holidays; however, they are significantly inferior to the New Year in the share of posts with speech acts and congratulations. Generally, this corresponds to the data of VCIOM (2018), which reports that these two holidays are celebrated by 84% and 88% of Russians, respectively.
– Victory Day is one of the most popular holidays to follow after the New Year, Defender of the Fatherland Day, and International Women’s Day. This is partially consistent with the results of the VCIOM (2018) survey, which showed that Victory Day is one of the most popular holidays; however, according to their results, it is more popular than Defender of the Fatherland Day and International Women’s Day.
– Russia Day is not among the 10 most popular holidays on Russian-speaking Twitter, ranking 11th in the overall ranking. In the VCIOM (2018) survey, this holiday is also one of the least celebrated compared to other official holidays.
– Union Day is ranked even lower that the Russia Day, which perfectly aligns with the VCIOM (2018) survey.
– International Workers’ Day is not really popular on Russian-speaking Twitter, and in comparison with other non-holiday days, it has only a slight increase in the share of speech acts and congratulations, ranking only 15 out of 22. Young Russians, who predominate on the analysed Twitter sample, avoid any ideological interpretation of the meaning of this holiday, more often perceiving May 1 as just an additional day off (VCIOM, 2004). Moreover, over a quarter of a century, the number of Russians who plan to celebrate Labor Day has been gradually decreasing (VCIOM, 2019b).
– The official holiday of Christmas, which is celebrated on January 7, was at the very bottom of the ranking (20 out of 22). At the same time, the VCIOM (2018) survey showed that Christmas in one of the main holidays in Russia which is being celebrated by 77% of population. We hypothesise that part of this can be explained by the fact that the younger age group that dominates Twitter is less religious than the older age group (Kofanova & Mchedlova, 2010). At the same time, Christmas, which is also celebrated by some on December 25, while not being an official holiday, ranked higher in the ranking and took 9th place. However, the high value on this day can also be caused by the approach of the main holiday of the year: the New Year.

- **Foreign holidays.** Halloween on Russian-speaking Twitter is one of the most popular foreign holidays, and is in 9th place among all days of 2020 in terms of the share of greetings and speech acts. This finding is not consistent with the results of VCIOM (2018), but we assume that this is due to the fact that Twitter is dominated by a younger age group that is more inclined to celebrate Halloween (VCIOM, 2019a), whereas the VCIOM (2018) survey provides a representative sample of the Russia population. Other foreign holidays, such as Chinese New Year and St. Patrick’s Day, are practically not celebrated on Russian-speaking Twitter: the share of greetings and speech act posts in 2020 these days was not only extremely small, but was generally lower than the yearly average (275th and 335th place, respectively). Overall, Chinese New Year and St. Patrick’s Day have not gained popularity on Russian-language Twitter and sit at the bottom of the overall rankings. This finding aligns with the results reported by VCIOM (2018): these holidays have not yet become widespread in Russia and they are celebrated by only 3–5% of the population, while 95–96% of respondents claim that they do not celebrate them.
- **Other common holidays.** Among other holidays, one of the most popular is Knowledge Day, which occupies the penultimate place in the top 10 most popular holidays.

Interestingly, on average, women are more likely to post greetings and speech acts tweets, but this pattern is observed not only on holidays, but also on ordinary days.

## Additional Information and Declarations

### Data Availability

The following information was supplied regarding data availability: The code for OSWB index construction is available at GitHub: https://github.com/sismetanin/oswb-russian-tweets; Sergey Smetanin. (2022). sismetanin/oswb-russian-tweets: v1 (Version v1). Zenodo. https://doi.org/10.5281/zenodo.7306128. The sentiment classification model is available at HuggingFace: https://huggingface.co/sismetanin/ruroberta-ru-rusentitweet; https://doi.org/10.57967/hf/0103. The code and the model for gender classification are available at GitHub: https://github.com/sismetanin/gender-by-name-vk; Sergey Smetanin. (2022). sismetanin/gender-by-name-vk: v1 (Version v1). Zenodo. https://doi.org/10.5281/zenodo.7306126. The following publicly available data were used in this study: - RuSentiTweet is available at GitHub: https://github.com/sismetanin/rusentitweet; Sergey Smetanin. (2022). sismetanin/rusentitweet: v1 (Version v1). Zenodo. https://doi.org/10.5281/zenodo.7314509. - Twitter Stream Grab is available at Internet Archive: https://archive.org/details/twitterstream.
